# Aortic valve stenosis awareness in Austria—results of a nationwide survey in 1001 subjects

**DOI:** 10.1007/s10354-019-00708-2

**Published:** 2019-09-20

**Authors:** Christian Hengstenberg, Martin Thoenes, Peter Bramlage, Jolanta Siller-Matula, Julia Mascherbauer

**Affiliations:** 1 Department of Internal Medicine, Division of Cardiology, Medical University Vienna, Währinger Gürtel 18–20, 1090 Vienna, Austria; 2 Edwards Lifesciences, Nyon, Switzerland; 3 Institute for Pharmacology and Preventive Medicine, Cloppenburg, Germany

**Keywords:** TAVI, TAVR, Heart valve disease, Aortic valve stenosis, Survey, Austria, TAVI, TAVR, Herzklappenerkrankung, Aortenklappenstenose, Umfrage, Österreich

## Abstract

Despite the prognostic significance of severe aortic valve stenosis, knowledge is limited in the general population. To document the status quo for Austria, knowledge about valvular heart disease/aortic valve stenosis was documented in 1001 participants >60 years of age. 6.7% of respondents were knowledgeable of aortic valve stenosis, with 1.6% being concerned about the condition (24.1% cancer, 18.8% Alzheimer’s disease, 15.1% stroke). 29.5% were familiar with valvular heart disease (76.7% heart attack, 36.9% stroke). Only 1/3 reported auscultation by their general practitioner (GP) at least every third visit. Typical symptoms of aortic valve stenosis were likely to be reported by 50%. After exposure to further information on aortic valve stenosis, only 20% reported to be more concerned and ready to obtain more disease-related information. Awareness of surgical and catheter-based treatment options was claimed by 77% of respondents. Awareness campaigns on valvular heart disease are warranted to improve patient care in Austria.

## Zusammenfassung

Trotz der prognostischen Relevanz einer schweren Aortenklappenstenose ist die Kenntnis in der Allgemeinbevölkerung begrenzt. Im Rahmen einer Umfrage wurde der Wissenstand bezüglich Herzklappenerkrankungen/Aortenstenose in der österreichischen Bevölkerung (>60 Jahre alt) untersucht. Von den befragten 1001 Teilnehmern waren 6,7 % mit dem Krankheitsbild Aortenklappenstenose vertraut, wobei lediglich 1,6 % hierdurch beunruhigt waren (24,1 % Krebserkrankungen, 18,8 % Morbus Alzheimer, 15,1 % Schlaganfall). Es gaben 29,5 % an, mit Herzklappenerkrankungen vertraut zu sein (76,7 % Herzinfarkt, 36,9 % Schlaganfall). Lediglich 1/3 erhielt durch den Hausarzt eine Herzauskultation bei mindestens jedem dritten Arztbesuch. Lediglich 50 % würden bei Bestehen von typischen Symptomen diese beim Hausarztbesuch erwähnen. Nach Bereitstellung von klinischen Informationen zur Aortenklappenstenose gaben lediglich 20 % an, nunmehr stärker beunruhigt zu sein und über Herzklappenerkrankungen mehr Informationen einholen zu wollen. Bezüglich verfügbarer therapeutischer Optionen waren insgesamt 77 % der Befragten der chirurgische sowie der kathetergestützte Klappenersatz bekannt. Die erhobenen Daten zeigen die Notwendigkeit einer verstärkten Aufklärung zum Thema Herzklappenerkrankungen/Aortenklappenstenose in Österreich auf.

## Introduction

**Fig. 1:**
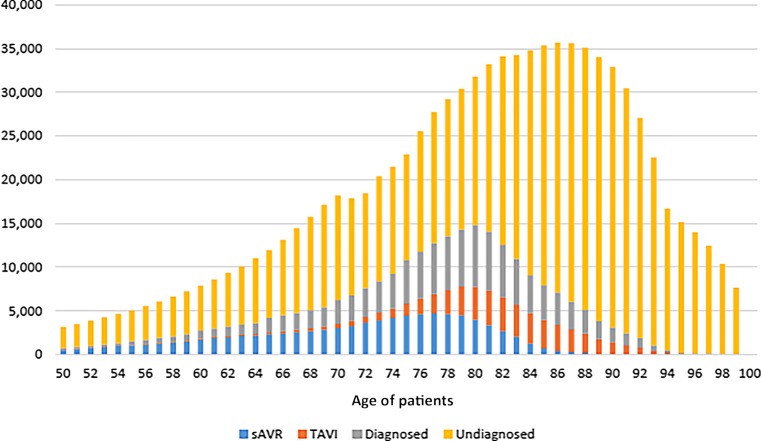
Estimated number of patients diagnosed with severe symptomatic aortic stenosis in the European Union by age and the proportion of patients remaining undiagnosed or undergoing sAVR or TAVI. *sAVR* surgical aortic valve replacement, *TAVI* transcatheter aortic valve implantation. (© Republished with permission of AME Publishing Company, from Thoenes et al. [10]; permission conveyed through Copyright Clearance Center, Inc. This figure is not included under the Creative Commons CC BY license of this publication)

Valvular heart disease represents a progressive and potentially life-threatening condition once the disease has progressed to a severe stage and no valve replacement therapy is performed [1]. According to recently published data, 12% of subjects >75 years old suffer from some degree of degenerative aortic valve stenosis and 3.4% from severe aortic valve stenosis [2]. The occurrence of typical symptoms such as dizziness/syncope, angina, or dyspnea should be considered the appropriate timepoint to consider valve replacement therapy (surgical replacement or transcatheter aortic valve implantation [TAVI]) for patients at a severe stage [3]. However, the emergence of symptoms not only represents an arbitrary timepoint for considering therapy, but symptoms also have to be considered unspecific, as they can be attributed to various health conditions in an elderly patient population [4, 5]. As symptoms are precedented by a variable time period of slow disease progression, patient awareness on valvular heart disease and associated symptoms appears to be key to identifying affected patients early and initiating timely and safe treatment in order to prevent irreversible myocardial damage and premature death. However, only a minority of patients suffering from severe aortic valve stenosis actually receive valve replacement therapy, mainly due to underdiagnosis of their condition (Fig. 1). Furthermore, the recently conducted IMPULSE study indicates that the vast majority of patients with aortic valve stenosis are diagnosed late and at an advanced age, which might limit potential treatment options due to an elevated or even prohibitive perioperative risk level [6]. In light of the tremendous importance of a timely diagnosis of valvular heart disease, a nationwide health survey was conducted in Austria in order to explore the level of awareness on the subject of aortic valve stenosis in the elderly population.

## Methods

A representative sample of individuals was selected and contacted by e‑mail. The sample consisted of males and females aged >60 years across nine regions in Austria. Survey invitations were emailed directly to panel members aged >60 years, with mean survey length of 3 min, and the full sample size of 1001 completes achieved within 5 days. Suitable panelists were identified through profiling questions asked when individuals first joined the panel via online registration. The selected cohort consisted of a representative sample of participants from urban and rural areas of Austria, classified as city/large town, small town, or villages/smaller settlements. This was self-defined by the respondents and they were asked which best suits where they live most of the time within 9 regions of Austria (Burgenland, Kärnten, Niederösterreich, Oberösterreich, Salzburg, Steiermark, Tirol, Vorarlberg, Wien). Panelists were put through a double opt-in procedure when they joined, ensuring they are eligible to receive invites when a survey matches the criteria they have entered.

The survey consisted of eight questions related to the awareness of aortic valve stenosis or valvular heart disease; the level of familiarity with several cardiac diseases; the level of concern related to other health conditions; the interactions with their general practitioner with regards to symptom reporting, auscultation practice, and preferences for particular health checks; and their level of knowledge related to several options for heart valve disease treatment. Furthermore, survey participants were provided with basic information on aortic valve stenosis (pathophysiology, symptoms, prognosis, and therapeutic options) and asked about their reaction and potential actions (Table 1). The survey was conducted by CensusWide (London).

**Table 1:** Questionnaire

Questions (Q)	Categories (C)
Q1: Do you know what aortic valve stenosis is?	None
Q2: Which of the following health conditions concerns you most?	C1: Infections C2: Heart valve disease C3: Parkinson’s disease C4: Arthritis C5: Respiratory disease C6: Diabetes C7: Heart attack C8: Stroke C9: Alzheimer’s disease C10: Cancer C11: None of the above
Q3: Which of the following heart conditions are you most familiar with?	C1: Rheumatic heart disease C2: Arrhythmia C3: Coronary heart disease C4: Angina C5: Congestive heart failure C6: Heart valve disease C7: Sudden cardiac death C8: Heart attack C9: All of the above C10: None of the above
Q4: When you visit your general practitioner, how often does he/she check your heart with a stethoscope?	C1: Every visit C2: Every second visit C3: Every third visit or less C4: Rarely C5: Never C6: I do not have/have not visited a general practitioner
Q5: How likely are you to report any of the following symptoms to your GP? (scale 1—extremely unlikely to 5—extremely likely)	C1: Chest tightness C2: Chest pain C3: Palpitations C4: Fatigue C5: Reduced physical activity C6: Shortness of breath C7: Feeling faint C8: Fainting upon exertion C9: Feeling older than your age
Q6: Are you more concerned about the disease and if so, what will you do as a result?	C1: I am not more concerned C2: I am more concerned and will seek more information on the subject C3: I am more concerned, but I have no plans to do anything C4: I am more concerned and I recognize the symptoms in myself C5: I am already aware of aortic stenosis
Q7: As part of the regular health checks for over 65s, which of the following should, in your view, GPs check for (ranking in order of priority)?	C1: Heart valve disease C2: Diabetes C3: Blood pressure C4: Osteoporosis C5: Alzheimer’s disease C6: Cholesterol
Q8: Which of the following therapies for heart valve disease are you aware of?	C1: Surgical valve replacement C2: Transcatheter valve replacement C3: Drug therapy C4: None of the above

Data were analyzed using descriptive statistics and presented as absolute values and frequencies (%). Comparison between different groups were carried out using Fisher’s exact or chi-square test, as appropriate. In all cases, a two-tailed *p*-value of <0.05 was considered statistically significant. All statistical tests were performed using IBM SPSS Statistics software version 24.0 (IBM Corporation, Armonk, NY, USA).

## Results

A total of 1001 subjects completed the online survey between 3 August 2018 and 8 August 2018, of whom 602 were male and 399 female. 44% of participants were aged 60–64 years, 28.5% aged 65–69 years, 20% aged 70–74 years, 6.3% aged 75–79 years, and 1% were more than 80 years old. Almost half of the subjects were enrolled in cities/larger towns (*n* = 477), 202 subjects in smaller towns, and 322 participants in rural areas (village/smaller settlement).

### Question 1

**Fig. 2:**
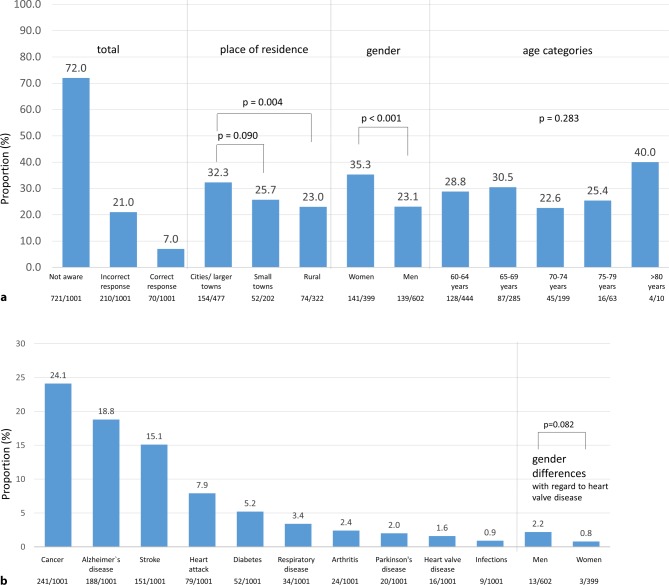
Aortic valve stenosis awareness (**a** Question 1); level of concern related to various diseases (**b** Question 2)

When asked about awareness of aortic stenosis, 72% of participants were not aware, whereas 28% claimed knowledgeable about the condition. Of the latter participants, 21% provided an incorrect response related to aortic valve stenosis, and in only 7% was an appropriate response provided. The highest awareness rates were documented in cities/larger towns, with 32.3% of participants being aware of aortic valve stenosis, whereas the lowest level was recorded in rural areas with 23% (*p* = 0.004). The highest awareness rate was observed in Klagenfurt (34.9%), whereas only 8.7% of participants from Leoben were aware of the condition. More women indicated awareness of aortic valve stenosis than men (35.3% vs. 23.1%; *p* < 0.001) and awareness rates ranged from 22.6% in the age group of 70–74 years up to 40% in the age group of >80 years, with no apparent trend across age groups (*p* = 0.283; Fig. 2a).

### Question 2

Participants were further asked about their level of concern related to various, mostly chronic, health conditions, including heart valve disease. Amongst 11 diseases, cancer represented the number one concern for 24.1% of respondents, followed by Alzheimer’s disease (18.8%) and stroke (15.1%). 2–8% of participants were mostly concerned about conditions such as heart attack, respiratory disease, arthritis, and Parkinson’s disease. Heart valve disease ranked as the second lowest disease of concern, with only 1.6% of respondents. Overall, 18.8% were not concerned about any of the diseases mentioned. No major differences between men and women were detected (*p* = 0.082). Heart valve disease ranked equally low in all age groups (below 2%), but in alignment with the high level of disease awareness, the highest in participants >80 years (30%) (Fig. 2b). Interestingly, 7.4% of participants in St. Pölten were concerned about heart valve disease, the highest proportion in the overall population.

### Question 3

**Fig. 3:**
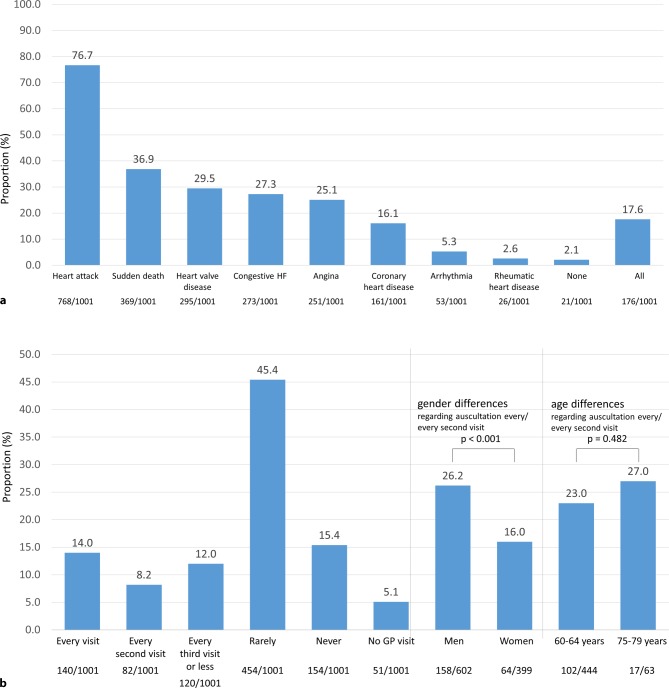
Level of familiarity with particular heart diseases (**a** Question 3); frequency of auscultation by general practitioners (**b** Question 4). *HF* heart failure, *GP* general practitioner; multiple options possible, wherefore values do not sum up to 100%

Looking into familiarity with particular heart diseases, the vast majority of respondents were familiar with heart attacks (76.7%), followed by sudden cardiac death (36.9%), heart valve disease (29.5%), congestive heart failure (27.3%), and angina (25.1%). 17.6% of respondents were somehow familiar with all categories of diseases and only 2.1% with none of them. There was no gender- or age-related difference with respect to the distribution recorded (Fig. 3a).

### Question 4

More than half of participants reported no auscultation or only at rare occasions by their general practitioner (61%), whereas in 14% of participants, a stethoscope is reportedly used at every consultation. 5.1% of participants reported no visits to their general practitioner. Interestingly, men appeared to be more frequently auscultated than women (26.2% vs. 16.0% at every/every second visit; *p* < 0.001) and overall auscultation frequency did not increase significantly with age (age categories 60–64 years vs. 75–79 years; *p* = 0.482; Fig. 3b).

### Question 5

When asked about the likelihood of reporting symptoms that could be potentially attributed to aortic valve stenosis, about 50% of participants would report chest tightness, chest pain, shortness of breath, feeling faint, or fainting on exertion to their general practitioner. Females are most likely to report fainting on exertion (37.1%), whereas men are most likely to report chest pain (29.2%). Feeling older than real age was associated with the lowest likelihood of being reported (Table 2).

**Table 2:** Question 5: How likely are you to report any of the following symptoms to your GP?

	Extremely unlikely (%)	Somewhat unlikely (%)	Neither likely nor unlikely (%)	Somewhat likely (%)	Extremely likely (%)
Chest tightness	22.2	12.9	14.6	27.4	23.0
Chest pain	20.8	13.4	12.6	24.3	29.0
Palpitations	19.0	16.9	23.2	25.5	15.5
Fatigue	16.2	15.0	21.1	33.6	14.2
Reduced physical activity	15.2	16.6	23.2	31.5	13.6
Shortness of breath	15.9	12.4	18.7	32.7	20.4
Feeling faint	20.0	11.0	15.8	24.8	28.5
Fainting upon exertion	25.9	11.0	14.8	16.2	32.2
Feeling other than your age	28.7	18.6	26.0	17.1	9.7

Scale: 1 “extremely unlikely” to 5 “extremely likely”

All values given as percentage

### Question 6

**Fig. 4:**
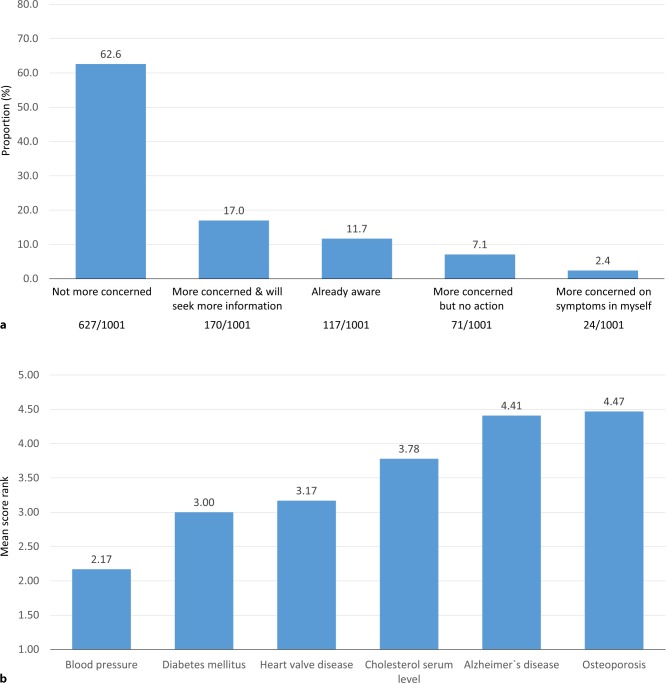
Change in perception of aortic valve stenosis after provision of basic disease-related information (**a** Question 6), suggested health checks for subjects >65 years of age (**b** Question 7); six different health conditions were provided for suggested health checks, ranking from 1–6 (highest–lowest priority) and mean score ranks are presented for each condition

Patients were provided with a short summary of pathophysiology, symptoms, prognosis, and the effect of valve replacement therapy in aortic valve stenosis. In the majority of patients, the information provided did not result in an increase in the level of concern about the condition (62.6%). Furthermore, 17% of participants reported being more concerned and interested in seeking more information, and 2.4% were more concerned as they recognized symptoms in themselves. However, 11.7% of participants reported to be already aware of aortic valve stenosis and 7.1% were more concerned but were not planning to take any particular action. There was no significant gender difference with respect to the observed change in perception of aortic valve stenosis (Fig. 4a).

### Question 7

All participants were proposed six different diseases for suggested regular screening in patients over 65 years and a ranking from 1–6 had to be applied for each (1 being the most important). The highest priority (lowest score rank) was recorded for blood pressure (mean score rank 2.17), followed by diabetes (3.00), heart valve disease (3.17), cholesterol (3.78), Alzheimer’s disease (4.41), and osteoporosis (4.47; Fig. 4b).

### Question 8

**Fig. 5:**
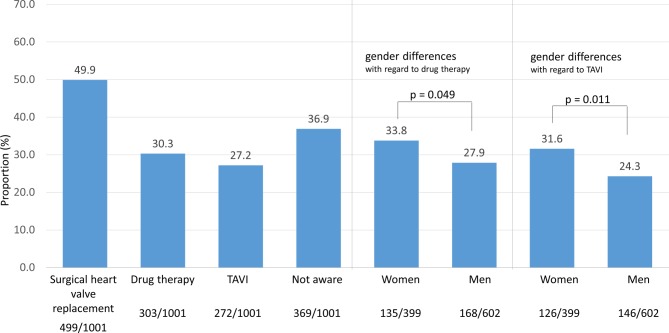
Awareness rates for different therapeutic options for heart valve disease (Question 8).*TAVI* transcatheter aortic valve replacement

Ultimately, knowledge on existing options for treating heart valve disease was explored in all survey participants. Half of all respondents were aware of open heart valve replacement surgery (49.9%), 30.3% of drug therapy, and 27.2% of transcatheter valve implantation. More than one third of participants were not aware of any of the abovementioned treatment options (36.9%). Women appeared to be more aware than men about drug therapy (33.8% vs. 27.9%; *p* = 0.049) and transcatheter valve implantation (31.6% vs. 24.3%; *p* = 0.011). Awareness rates for different treatment options did not appear to change with increasing age (Fig. 5).

## Discussion

The burden of valvular heart disease is increasing due to an increased life expectancy in the elderly population [2, 7]. Today, 18.6% (1.6 million) of inhabitants of Austria are 65 years or older, a number which is projected to rise to 23% in 2030 [8]. Based on an estimated number of Austrian citizens aged >75 years of 1 million, about 120,000 inhabitants could be affected by aortic valve stenosis (any degree) and 30–40,000 by severe aortic stenosis [2]. Like in most other Western countries, cardiovascular disease represents the most frequent cause of death in Austria, with about 33,000 patients affected in 2017 [9]. All of the above indicate a significant burden of valvular heart disease in Austria. As the majority of patients affected by aortic valve stenosis do not receive treatment due to underdiagnosis of their condition, patient awareness appears to be of utmost importance to improve care of valvular heart disease [10]. Our survey indicates a low level of patient knowledge on aortic valve stenosis in Austria (Fig. 2a), confirming data from other recently published survey results [11]. Furthermore, valvular heart disease awareness did not appear to increase with age and was higher in women, indicating insufficient medical education on age-dependent crucial health conditions on the one hand, and potential gender differences in health consciousness in women as compared to men [12, 13]. Despite the fact that cardiovascular disease represents the most important cause of death in Austria, cardiac conditions such as heart attack and heart valve disease ranked low with regards to the level of concern, which is mostly probably due to a high level of public presence (media) of other health conditions, such as cancer, Alzheimer’s disease, and stroke, and the perception of their relevant impact on individual’s mortality and disability in conjunction with the promotion of early screening campaigns [14, 15]. When asked about particular cardiovascular diseases, the vast majority of respondents were familiar with heart attack, but valvular heart disease ranked number three, confirming the survey data on aortic valve stenosis awareness rates (Fig. 2a). Despite its limitations with regards to sensitivity and specificity to diagnose valvular heart disease, chest auscultation represents an important diagnostic measure to support the diagnosis of suspected aortic valve stenosis [16, 17]. Recently published data confirm a low rate of auscultation in Europe and therefore suggest that a significant proportion of patients with aortic valve stenosis may not be referred for echocardiography [11, 18, 19]. In line with these findings, our data confirm a low frequency of auscultation in Austria, where the auscultation rate does not appear to increase with age (Fig. 3b). This might be explained by the impact of patient age on the patient–general practitioner interaction, where elderly patients tend to receive less counseling, are asked fewer questions, and are more often monitored for treatment compliance only. Furthermore, interactions of patients with their general practitioner are usually short, and a skillful auscultation mandates appropriate preparation [20, 21]. As symptoms not only represent the appropriate timepoint for aortic valve replacement in severe aortic valve stenosis but also the reason to visit a physician and initiate diagnostic measures, patient awareness of symptoms and readiness to report them to their general practitioners are crucial. As the likelihood of symptom reporting was low overall, our findings highlight the importance of patient education on typical disease symptoms in order to enable an early diagnosis and timely treatment of aortic valve stenosis (Table 2). As a continuous approach to medical advice on health conditions, our data do not confirm an immediate effect on the level of concern after the provision of disease-related information (Fig. 4a). It has to be mentioned, however, that the majority of participants included in this survey did not report typical symptoms of heart valve disease or the diagnosis of a heart condition.

However, valvular heart disease ranked as the third most important disease for suggested routine health checks after elevated blood pressure and diabetes, a finding which might be explained by the low level of invasiveness of diagnostic measures for these two conditions, but potentially also by the educational nature of the survey itself, prompting patients to elevate the importance of valvular heart disease in their perception (Fig. 4b).

Furthermore, the data also confirm a higher awareness of surgical compared to catheter-based treatment options, despite the fact that the latter were introduced more than 10 years ago and have gained increasing attention, also in the public media. The data from the present online survey in the elderly population in Austria confirm the need for more medical education measures for primary care physicians and patients related to valvular heart disease in order to increase diagnosis and treatment rates. Finally, it has to be mentioned that a certain level of patient selection bias cannot be excluded, as all survey participants had online access, which might indicate a higher level of access to disease-related information. However, one also needs to consider that disease awareness appeared to be low and online access is available to the majority of the population.

## References

[1] Kapadia SR, Leon MB, Makkar RR, Tuzcu EM, Svensson LG, Kodali S, Webb JG, Mack MJ, Douglas PS, Thourani VH, Babaliaros VC, Herrmann HC, Szeto WY, Pichard AD, Williams MR, Fontana GP, Miller DC, Anderson WN, Akin JJ, Davidson MJ, Smith CR, investigators Pt (2015). 5-year outcomes of transcatheter aortic valve replacement compared with standard treatment for patients with inoperable aortic stenosis (PARTNER 1): a randomised controlled trial. Lancet.

[2] Osnabrugge RL, Mylotte D, Head SJ, Van Mieghem NM, Nkomo VT, LeReun CM, Bogers AJ, Piazza N, Kappetein AP (2013). Aortic stenosis in the elderly: disease prevalence and number of candidates for transcatheter aortic valve replacement: a meta-analysis and modeling study. J Am Coll Cardiol.

[3] Baumgartner H, Falk V, Bax JJ, De Bonis M, Hamm C, Holm PJ, Iung B, Lancellotti P, Lansac E, Rodriguez Munoz D, Rosenhek R, Sjogren J, Tornos Mas P, Vahanian A, Walther T, Wendler O, Windecker S, Zamorano JL, Group ESCSD (2017). 2017 ESC/EACTS Guidelines for the management of valvular heart disease. Eur Heart J.

[4] Maarsingh OR, Dros J, Schellevis FG, van Weert HC, van der Windt DA, ter Riet G, van der Horst HE (2010). Causes of persistent dizziness in elderly patients in primary care. Ann Fam Med.

[5] Frese T, Mahlmeister J, Deutsch T, Sandholzer H (2016). Reasons for elderly patients GP visits: results of a cross-sectional study. Clin Interv Aging.

[6] Frey N, Steeds RP, Serra A, Schulz E, Baldus S, Lutz M, Pohlmann C, Kurucova J, Bramlage P, Messika-Zeitoun D (2017). Quality of care assessment and improvement in aortic stenosis—rationale and design of a multicentre registry (IMPULSE). Bmc Cardiovasc Disord.

[7] Nkomo VT, Gardin JM, Skelton TN, Gottdiener JS, Scott CG, Enriquez-Sarano M (2006). Burden of valvular heart diseases: a population-based study. Lancet.

[8] Statistik Austria (2999). Bevölkerung 2017.

[9] Statistik Austria (2999). Absolute und relative Häufigkeiten der Gestorbenen sowie durchschnittliches und empirisches Sterbealter nach Todesursachen und Geschlecht 2017.

[10] Thoenes M, Bramlage P, Zamorano P, Messika-Zeitoun D, Wendt D, Kasel M, Kurucova J, Steeds RP (2018). Patient screening for early detection of aortic stenosis (AS)-review of current practice and future perspectives. J Thorac Dis.

[11] Gaede L, Di Bartolomeo R, van der Kley F, Elsasser A, Iung B, Mollmann H (2016). Aortic valve stenosis: what do people know? A heart valve disease awareness survey of over 8,800 people aged 60 or over. EuroIntervention.

[12] Girois SB, Kumanyika SK, Morabia A, Mauger E (2001). A comparison of knowledge and attitudes about diet and health among 35- to 75-year-old adults in the United States and Geneva, Switzerland. Am J Public Health.

[13] Ek S (2015). Gender differences in health information behaviour: a Finnish population-based survey. Health Promot Int.

[14] Alzheimer’s association. www.alz.org/abam. Zugegriffen: 19. January 2019.

[15] World Alzheimer’s Month. www.worldalzmonth.org. Zugegriffen: 19. January 2019.

[16] Bodegard J, Skretteberg PT, Gjesdal K, Pyorala K, Kjeldsen SE, Liestol K, Erikssen G, Erikssen J (2012). Low-grade systolic murmurs in healthy middle-aged individuals: innocent or clinically significant? A 35-year follow-up study of 2014 Norwegian men. J Intern Med.

[17] Myerson S, Prendergast B, Gardezi S, Prothero A, Kennedy A, Wilson J (2017). GP auscultation for diagnosing valvular heart disease. Heart.

[18] Bouma BJ, van der Meulen JH, van den Brink RB, Arnold AE, Smidts A, Teunter LH, Lie KI, Tijssen JG (2001). Variability in treatment advice for elderly patients with aortic stenosis: a nationwide survey in The Netherlands. Heart.

[19] Webb J, Thoenes M, Chambers JB (2014). Identifying heart valve disease in primary care: differences between practice in Germany, France and the United Kingdom. Eur. Eur. J. Cardiovasc. Med..

[20] Chizner MA (2008). Cardiac auscultation: rediscovering the lost art. Curr Probl Cardiol.

[21] Callahan EJ, Bertakis KD, Azari R, Robbins JA, Helms LJ, Chang DW (2000). The influence of patient age on primary care resident physician-patient interaction. J Am Geriatr Soc.

